# Toward Shape Memory Alloy Actuation in Exoskeletons: A Large Language Model-Assisted Fuzzy Control Architecture with a Servo-Based Proof of Concept

**DOI:** 10.3390/ma19214613

**Published:** 2026-10-30

**Authors:** Jakub Kopowski, Agnieszka Osińska, Maciej Gniadek, Dariusz Mikołajewski, Izabela Rojek

**Affiliations:** Faculty of Computer Science, Kazimierz Wielki University, Chodkiewicza 30, 85-064 Bydgoszcz, Poland; jakub.kopowski@ukw.edu.pl (J.K.); agnieszka.osinska@ukw.edu.pl (A.O.); maciej.gniadek@ukw.edu.pl (M.G.); dariusz.mikolajewski@ukw.edu.pl (D.M.)

**Keywords:** shape-memory alloys, servo drives, exoskeletons, Sugeno fuzzy inference, large language models, cyber–physical systems, command interpretation

## Abstract

**Highlights:**

- A local LLM converted natural-language commands into structured servo target positions.
- Fixed-step and zero-order Sugeno motion-profile generators were implemented and functionally tested.
- The layered architecture separates semantic interpretation from actuator-specific motion execution.

**Abstract:**

Shape-memory alloys (SMAs) offer low mass, compact integration, quiet operation, and high work density, but their hysteresis and coupled thermal–mechanical behavior complicate repeatable control. This study develops a modular architecture in which natural-language command interpretation is separated from motion-profile generation and from actuator-specific models and controllers. The implemented pilot path was evaluated on a five-servo robotic hand, while the common comparison layer defines interfaces for technology-specific numerical models reserved for the next implementation stage. A locally executed large language model (LLM) converted user commands into port:angle pairs, and the same semantic front-end was coupled to either a fixed-step or zero-order Sugeno motion-profile generator. The semantic layer was evaluated using 60 recorded commands: 27 supported commands and 33 commands requiring rejection. Exact target generation was obtained in 25 of 27 supported cases (92.6%), while 17 of 33 invalid or unsupported commands were correctly rejected (51.5%), giving a strict overall result of 42 of 60 cases (70.0%). Direct observation confirmed the expected physical outcome in 23 of 27 supported cases (85.2%). The mean local response time was 25.30 s, the median was 16.71 s, and the 95th percentile was 66.73 s, confirming that the LLM is suitable only for the supervisory semantic layer. Because the pilot setup did not use an external position sensor, the fuzzy module is interpreted as a command-profile generator rather than a closed-loop position controller. The results validate the layered interface and establish an experimental and computational basis for a subsequent quantitative comparison of SMA and servo actuation.

## 1. Introduction

Exoskeletons are cyber–physical systems that interact directly with humans; consequently, their proper operation depends not only on their mechanical design and the type of actuation employed but also on how the system’s dynamics are represented, how sensory data is processed, how trajectories are generated, and how control is implemented in real time [1,2,3,4,5,6,7,8,9,10,11,12,13,14,15,16,17,18,19,20,21,22]. From the perspective of technical informatics, a significant challenge lies in developing a computational architecture capable of managing actuators characterized by diverse physical properties, constraints, and time scales. These differences influence the model structure, the selection of state variables, the controller update frequency, the handling of constraints, and computational costs. Simultaneously, the development of large language models (LLMs) enables the introduction of a supervisory semantic layer designed to interpret user commands and convert them into structured reference parameters that are independent of the specific actuation technology used [23,24,25,26,27,28,29,30,31,32,33,34,35,36,37,38]. In this setup, the LLM does not replace the deterministic controller or participate directly in the high-speed control loop; instead, it works in conjunction with a mechanism that validates the correctness and safety of the generated parameters. Thus, there is a need to develop a multi-layered architecture that integrates numerical models, intelligent control algorithms, and a natural language user interface, while ensuring predictable and safe movement execution.

The actuation system is of particular importance in an exoskeleton, as its properties influence the mass and inertia of the wearable component, movement execution accuracy, interaction safety, and user comfort. An actuator cannot be evaluated solely based on maximum torque or power; factors such as dynamic bandwidth, mechanical compliance, back drivability, energy consumption, noise, thermal constraints, and the ability to control the force and energy transmitted to the human user must also be considered. Most modern designs employ electric servo-actuators coupled with gearboxes, cable-driven mechanisms, or elastic elements. These systems offer high accuracy, repeatability, and relatively wide bandwidth, and can be controlled using established current, speed, and position regulation schemes. However, gearboxes increase mass, friction, backlash, and reflected inertia, while Bowden cables and elastic actuators introduce additional compliance and nonlinearity. Thus, while the servo-actuator serves as a suitable baseline, it does not resolve all the challenges associated with lightweight wearable devices [1,2,3,39,40,41,42,43,44,45,46,47,48,49,50,51,52,53,54,55,56,57,58,59,60].

Shape memory alloys—specifically NiTi-based materials—represent an alternative actuation technology in which phase transformation can be harnessed to generate force and displacement. Shape memory alloy (SMA) actuators offer low mass and volume, operate silently, and can be integrated into flexible and textile-based exoskeleton components. Their high work density and ability to produce movement functionally similar to muscle contraction make this technology attractive for wearable robotics. The literature describes solutions for assisting the movement of the elbow, wrist, hand, shoulder, and lower limb, as well as antagonistic, textile, and hybrid designs [61,62,63,64,65,66,67,68,69,70,71,72,73,74,75,76,77,78,79,80,81,82]. However, these design advantages come with challenges such as hysteresis, limited useful wire strain, thermal inertia, slow cooling, performance dependence on temperature and stress, and property degradation due to cyclic loading. Furthermore, achieving a low-mass active element does not automatically ensure high overall system efficiency, as the energy required for heating, cooling, and maintaining the material’s required state must also be taken into account.

The distinction between an SMA actuator and a servo drive is evident not only in their construction but, above all, in how they are represented computationally. A servo drive model typically encompasses electrical and mechanical dynamics, friction, inertia, transmission, and current–voltage limits. In contrast, an SMA actuator model requires the simultaneous consideration of electrical, thermal, phase-change, and mechanical phenomena. Current flowing through the active element causes Joule heating; temperature and stress influence the martensite and austenite fractions; and the phase transformation process determines strain, force, the elastic modulus, and resistance. Furthermore, the actuator’s state depends not only on the current input signal value but also on the history of loading and temperature. The literature presents models of varying complexity, ranging from one-dimensional constitutive models to thermodynamically consistent multidimensional models and solutions designed for finite element analysis [83,84,85,86,87,88,89,90,91,92,93,94,95,96,97,98,99,100]. However, control applications most often require reduced-order models that preserve key physical couplings while maintaining a computational cost that allows for repeated simulations, controller tuning, and robustness analysis [71,72,101,102,103,104,105,106,107,108,109,110,111,112].

SMA hysteresis is a nonlinearity with memory, meaning that for a given input value, the actuator can exist in different states depending on its prior thermal and mechanical trajectory. Neglecting this phenomenon can lead to tracking errors, phase shifts, increased control activity, and, under certain conditions, degraded system stability. Preisach and Prandtl–Ishlinskii models—along with their variants—enable the description and compensation of this dependency without fully modeling the material’s physics; however, they require parameter identification, and their parameters may vary with load, frequency, and cooling conditions [113,114,115,116,117,118,119,120,121,122]. While proportional–integral (PI) and proportional–integral–derivative (PID) controllers can ensure proper operation within a limited working range, their fixed settings do not always compensate for changes in plant parameters. Consequently, adaptive, robust, sliding-mode, model-free, and predictive control methods are being developed [123,124,125,126,127,128,129,130,131,132,133,134,135,136,137,138,139,140]. Fuzzy gain tuning and hybrid Preisach–fuzzy compensation address parameter variation and hysteresis without requiring a complete analytical model [141,142,143]. Neuro-fuzzy feedforward models and type-2 fuzzy controllers extend this idea toward data-driven adaptation and uncertainty representation [144,145]. Neural predictive control and ANFIS-based identification provide further data-driven alternatives [146,147], while LSTM, dynamic neural networks, and machine-learning models target temporal hysteresis and actuator dynamics [148,149,150]. Related implementations include fuzzy PI control, direct neural control, fuzzy PWM-PID control, and neural state estimation [151,152,153,154]. These approaches remain dependent on identification or training coverage. They can be complemented by using the SMA element’s electrical resistance to estimate its state, although this requires decoupling the effects of temperature, material phase, strain, and electrical connection conditions [155,156,157,158,159,160].

The practical rationale for this research stems from the need to determine whether the physical limitations of an alternative actuator can be partially compensated for through modeling and intelligent control strategies. If the effects of hysteresis, parameter variability, and thermal dynamics can be mitigated algorithmically, SMA technology could be suitable for applications where low mass, compactness, and silent operation are prioritized over high motion bandwidth. This does not imply that SMA technology should replace servo drives in every design; rather, it is crucial to define the scope of its appropriate application and explore the potential for combining both technologies in hybrid solutions. To date, research on SMA actuators and servo drives has typically involved varying loads, trajectories, frequencies, control structures, and performance criteria. Consequently, it is difficult to ascertain whether observed differences stem from the actuation technology itself, the chosen model, the controller employed, or the specific testing conditions [4,5,6,7,8,9,10,11,12,13,14,15,16,17,18,19,20,21,22,42,43,44,45,46,47,48,49,50,51,52,53,54,55,56,57,58,59,60,63,64,65,66,67,68,69,76,77,78,79,80,81,82]. A unified numerical environment helps address this issue by utilizing an identical motion task, comparable loads, consistent constraints, and the same set of performance metrics.

Such an architecture requires separating the task description from the method of its execution. The low-level layer should account for the physics of the specific actuator and implement control at a frequency dictated by its dynamics. The intermediate layer can handle trajectory generation, motion parameter limiting, and controller selection, while the high-level layer interprets the user’s goal. LLMs can support this latter function by decomposing high-level commands into tasks, selecting available functions, and generating structured execution parameters [23,24,25,26,27,28,29,30,31,32,34,35,36,37,38]. However, an LLM’s semantic knowledge does not guarantee the physical feasibility of a command. The model might generate parameters that are contradictory, exceed permissible ranges, or are incompatible with the exoskeleton’s current state. Additional challenges include variable latency, non-determinism, and the difficulty of formally guaranteeing safety [29,30,33]. Consequently, the LLM output should be constrained to a defined data schema and verified by a deterministic validator that checks the operating mode, range of motion, velocity, acceleration, torque, duration, and temperature. Only once accepted can the parameters be passed to the trajectory generator and the controller appropriate for the SMA or servo drive.

Despite advancements in SMA modeling, intelligent control methods, exoskeleton designs, and robotic language interfaces, these topics are typically analyzed in isolation. Existing research focuses on specific material properties [161,162,163,164], particular actuation types, individual controllers, or the use of LLMs for planning and communication. However, there is a lack of a unified architecture that would allow for a comparison between an SMA actuator and a conventional servo drive while performing the same exoskeleton task, using a consistent semantic layer and comparable evaluation criteria. Thus, the gap stems not from a lack of component models, but rather from a lack of integration and unified validation, as well as the separation of command interpretation from deterministic motion control.

The contribution of this work is the integration of three levels that are normally discussed separately: an actuator-independent semantic interface, an explicit motion-profile layer, and defined locations for actuator-specific numerical representations. The implemented pilot path verifies natural-language-to-servo data flow and compares two command-generation variants. The common comparison layer specifies how servo-drive and SMA modules can be connected to the same task description and evaluated using consistent outputs, while the technology-specific numerical models occupy clearly identified modules for the next implementation stage. This distinction is important: the LLM does not generate current, voltage, temperature, or torque commands and is not placed inside the fast control loop.

Accordingly, the aim is to establish a coherent computational framework for heterogeneous actuation in exoskeleton-oriented systems and to verify the supervisory path on an available servo-driven demonstrator. The study addresses the interpretation of user intent, structured reference generation, fixed and fuzzy motion-profile generation, local implementation requirements, and the boundary between semantic and execution layers. Neuro-fuzzy adaptation is retained as a prospective extension and is not reported as an implemented or experimentally validated controller.

## 2. State of the Art

Assessing the suitability of shape-memory alloys for exoskeletons requires simultaneously addressing material physics, actuation dynamics, control methods, and human–robot interaction architecture. The literature on these topics has evolved largely in isolation: SMA models focus on phase transformation and hysteresis; research on servo-actuators centers on rapid, repeatable motion control; exoskeleton studies prioritize safety and mechanical compliance; and the LLM literature deals with language interpretation and planning. The following synthesis integrates these fields from the perspective of technical informatics—encompassing unified numerical representation, intelligent control algorithms, structured software interfaces, and verifiable cyber–physical system architecture.

### 2.1. Exoskeleton Requirements and Selection of Actuation Technology

The actuation system of an exoskeleton operates in direct contact with the human body; therefore, it cannot be evaluated solely in terms of maximum torque or positional error. Equally important are the mass and inertia of the wearable components, power density, efficiency, bandwidth, mechanical compliance, back drivability, noise, thermal comfort, and the ability to limit the force and energy transmitted to the user. Furthermore, reviews of lower-limb exoskeletons indicate that the performance of individual designs is difficult to compare due to differences in tasks, users, trajectories, and performance metrics; the lack of standardized quantitative evaluation methods remains a challenge in this field [1,2,3,39,40,41,47,48,49,50,51,52,53,54,55].

Mature designs are dominated by geared electric motors, cable drives, and elastic actuators. Incorporating series elasticity reduces the impedance perceived by the user, improves impact resilience, and facilitates force control, yet it limits force bandwidth and necessitates a trade-off regarding stiffness selection [39]. Relocating the motor away from the limb segment via a Bowden cable reduces distal mass but introduces friction, backlash, and transmission hysteresis [40]. Newer nonlinear elastic actuators and quasi-direct-drive systems aim to further enhance transparency and interaction safety, confirming that the quality of an exoskeleton’s actuation depends on the integration of mechanics, sensing, and control, rather than the motor alone [41].

Shape-memory alloys offer a different set of trade-offs. The active element can be lightweight, compact, silent, and easily integrated into a soft structure; furthermore, high work density and the similarity of wire or spring contraction to muscle action make them well-suited for wearable applications [61,62]. The drawbacks include limited useful wire strain, the need for pre-loading or an antagonistic arrangement, hysteresis, temperature dependence, slower cooling, and property changes over repeated cycles. Consequently, SMA is not a universal substitute for servomotors. Its use is most justified in applications where reducing mass, volume, and noise outweighs the need for high-frequency cycling, whereas servomotors retain the advantage for rapid, sustained, high-torque movements [70,71,72,73,74,75,101,103].

### 2.2. Numerical Modeling of SMA Actuators

Modeling an SMA actuator requires coupling three domains: electrical, thermal, and mechanical. Electric current causes Joule heating; temperature and stress govern the martensite-austenite transformation; and phase fractions influence the elastic modulus, transformation stress, recoverable strain, and electrical resistance. The classical one-dimensional Liang–Rogers and Brinson models express these relationships using the martensite fraction as an internal variable [83,84]. The Brinson model distinguishes between temperature-induced and stress-induced martensite, thereby enabling the representation of both the shape memory effect and pseudoelasticity across a wider range of loading paths [83]. The thermodynamically consistent Boyd–Lagoudas models provide a more general framework for multiaxial and finite element analyses, though they increase the number of parameters and the computational cost of integrating the constitutive equations [85,86,87,88,89,90,91,92,93,94,95,96,97,98,99,100].

Model comparisons show that while their general forms may be similar, fundamental differences arise from phase transformation kinetics and the definition of internal variables [86]. Validation against thermomechanical tests demonstrated that the Tanaka and Liang–Rogers models can correctly reproduce pseudoelasticity under specific conditions but have limitations in describing the full shape memory effect; Brinson’s variant, featuring improved kinetics, proved to be more versatile [87]. Consequently, the choice of model should be dictated by the objective of the calculations. Material and local stress analysis justifies the use of a multidimensional model or finite element model (FEM), whereas extensive controller and robustness testing typically requires a reduced-order model that preserves the key physical couplings.

In lumped-parameter models, the heat balance of the wire can be expressed in a form that accounts for electrical power, convection, radiation, and phase-change heat:

m c_p_ Ṫ = i^2^R(T, ξ) − hA(T − T_a_) − εσSB A(T^4^ − T_a_^4^) − mLξ̇ (1)

where ξ represents the martensite fraction, and the convection coefficient h, resistance R, and ambient temperature T_a_ may vary with the operating state and conditions. Reducing the continuum equations to a small system of differential equations allows for the analysis of heating and cooling times, minimum power, and the influence of actuator geometry without costly field calculations [101]. Modular models combining the heat balance, constitutive equation, loading spring mechanics, and resistance facilitate the swapping of individual sub-models and their implementation in a control environment [102].

The assumption of uniform temperature is convenient, but for long wires, forced cooling, or intense heat exchange, it becomes a significant limitation of the reduced-order model [101]. Higher accuracy can be achieved by dividing the element into segments, at the cost of additional states and parameters. At the same time, the dynamic model must capture the effects of loading rate, arbitrary thermomechanical paths, and minor hysteresis loops [102,103,104]. Operating trajectories are not limited to the main hysteresis loop, and pulse-width modulation (PWM) control and frequent direction reversals generate smaller, subordinate loops. Accounting for these improves tracking performance, yet experiments also reveal drift and factors that are difficult to parameterize, meaning that a constant PWM duty cycle does not guarantee a constant position or force [105]. A model intended for comparing actuators should therefore preserve heating/cooling asymmetry, transformation history, and thermal constraints, while remaining computationally comparable to a servo-actuator model [106,107,108,109,110,111,112].

### 2.3. Hysteresis, Control, and State Estimation of SMA

Hysteresis is a nonlinearity with memory: for a given input value, the actuator state depends on the prior trajectory of temperature, stress, and strain. Neglecting it leads to open-loop control errors—manifesting as amplitude-dependent phase shifts and harmonic generation—and, in extreme cases, to a loss of loop stability [113]. Preisach and Prandtl–Ishlinskii models, along with their variants, describe the input–output relationship without fully capturing the material’s underlying physics. While useful for feedforward compensation, they require parameter identification, and their parameters may depend on load, frequency, and thermal conditions. The analytical invertibility of the generalized Prandtl–Ishlinskii model facilitates implementation; combining its inverse with a PI controller reduced overshoot and tracking error more effectively than a feedback loop alone [114,115,116,117,118,119,120,121,122].

Early tracking systems combined a hysteresis model in the feedforward path with a PID controller [123]. Nonlinear and variable-structure control enhanced robustness against model uncertainty but necessitated measures to limit control signal oscillations and overheating, as slow cooling introduces significant lag [124]. An architecture combining neural hysteresis compensation with robust sliding-mode control achieved accurate periodic trajectory tracking, with stability ensured by the feedback component rather than the data model itself [125]. Model-free control offers an alternative; in studies involving a spring actuator, it outperformed nonlinear PI control under varying loads and thermal disturbances while maintaining low computational cost [126]. Delay estimation and continuous terminal control reduce reliance on an explicit hysteresis model [127]; however, gain selection must still account for thermal parameter variations, the formal quantification of which can be based on Bayesian estimation [128,129,130,131,132,133,134,135,136,137,138,139,140].

Fuzzy control is attractive when precise system identification is impossible or parameters vary during operation. Self-tuning fuzzy PID, fuzzy PI, and fuzzy PWM-PID controllers adjust the control action using the error and its rate of change [141,151,153]. Hybrid Preisach–fuzzy compensation and fuzzy inverse Preisach models address hysteresis while reducing the burden of full analytical identification [142,143]. Neuro-fuzzy feedforward modeling and ANFIS-based identification introduce data-driven adaptation [144,147], whereas type-2 fuzzy logic represents uncertainty in membership functions [145]. Neural networks have also been applied to predictive control [146], temporal hysteresis modeling with LSTM networks [148], adaptive inverse and direct control [149,152], machine-learning-enhanced dynamic response modeling [150], and state estimation [154]. These benefits are conditional: performance depends on the coverage and quality of the training or identification data, and successful interpolation does not guarantee robustness outside the calibrated range.

Self-sensing offers an additional means of reducing mass and complexity. Wire resistance changes during the phase transformation and can be used to estimate position without an external sensor; experiments have demonstrated a strong correlation within specific temperature and load ranges [155]. Combining resistance-based estimation with major and minor loop compensation enables the miniaturization of the feedback system [156]. Modern one-dimensional convolutional neural network (1D-CNN) and LSTM models achieve comparable estimation accuracy, although 1D-CNNs may offer faster inference times on embedded platforms [157]. However, self-sensing does not eliminate ambiguity, as resistance depends simultaneously on temperature, phase, strain, and the properties of wires and contacts. Consequently, for exoskeleton applications, performance assessment following changes in load and cooling conditions is required, rather than mere calibration at a single operating point [158,159,160].

### 2.4. SMAs and Servo Drives in Wearable Robotics

Wearable prototypes confirm that SMA technology can provide a useful level of assistance while maintaining low weight. The elbow exoskeleton developed by Copaci et al. weighed approximately 0.6 kg and operated quietly, though the authors identified slow cooling as an unresolved functional issue [63]. A suit featuring SMA textile muscles and weighing less than 1 kg was capable of lifting loads of 2 kg and 4 kg, but increasing the load extended the time required to reach the target position to approximately 3 s [64]. A soft wrist system, with a wearable component weighing 151 g, generated up to 1.32 N·m of torque during flexion, while the spring-based active element achieved a contraction of up to 40% [65]. These results demonstrate a tangible design advantage, but also highlight a strong dependence of system dynamics on the load and transmission geometry.

Active cooling fundamentally alters the achievable bandwidth. In an elbow and pronation–supination support system, spring bundles were placed within a flexible cooling channel; a flexion torque exceeding 3 N·m was achieved, and trials were conducted with individuals who had suffered a stroke or spinal cord injury [66]. A biomimetic design featuring a rubber antagonistic element achieved a flexion range of approximately 0–80° at an SMA temperature of around 60–70 °C, a finding that underscores the necessity of explicitly monitoring thermal safety [67]. More recent work focuses on the hand and lower limb: a hand exoskeleton—both modeled and experimentally verified—demonstrates the potential for using resistance for control purposes [68], while a hybrid knee-ankle orthosis combines SMA technology with a brushless direct-current motor (BLDC) motor, treating these technologies as complementary rather than mutually exclusive [63,64,65,69,76,77,78,79,80,81,82].

The servo drive serves as a mature benchmark due to its rapid electrical dynamics and cascaded current, speed, and position loops. In permanent magnet synchronous motor (PMSM) drives, fuzzy sliding-mode control and membership function adaptation compensate for parameter and load variations [42], while recurrent neuro-fuzzy networks can learn the control law online while retaining a compensation term [43]. MPC improves tracking performance at the cost of computational effort; consequently, explicit solutions or shortened horizons are significant for high update frequencies [44]. Adaptive fuzzy compensation can be integrated into an existing P/PI cascade to estimate friction without redesigning the entire architecture [45], whereas tuning the current loop based on a discrete model demonstrates a timescale unattainable by thermal SMA—the response can be corrected within a few sampling periods [46,56,57,58,59,60].

Published results for SMA actuators and servo drives cannot be ranked directly because they were obtained with different masses, gear ratios, trajectories, cooling conditions, voltages, and safety criteria. A reliable comparison requires identical loading and reference profiles, shared mechanical limits, and the reporting of RMSE or MAE, phase lag, settling time, energy, peak power, control saturation, temperature, and computation time. Table 1 therefore summarizes the literature-derived variables and constraints that should be controlled or reported in a future comparison. It is an author synthesis of the physical and control requirements discussed in Section 2.1, Section 2.2, Section 2.3 and Section 2.4, not an experimental performance ranking.

### 2.5. Hierarchical Control and the Role of a Large Language Model

Exoskeleton control naturally follows a hierarchical structure. The high-level layer recognizes the intent, task, or movement phase; the intermediate layer determines the trajectory, torque, impedance, or assistance level; and the low-level layer regulates current, force, or position. In the LOPES system, impedance control enabled both patient-guiding and patient-following modes [4]. Neuro-fuzzy EMG processing has been employed for the adaptive interpretation of upper-limb intent [5], while reviews of sensing technologies highlight the absence of a single universal controller: feedforward signals from the neuromuscular system and feedback signals from the exoskeleton offer distinct advantages and entail different calibration requirements [6]. Multimodal architectures combine position control for passive training with impedance control for cooperative training [7], while neuro-fuzzy compensation and adaptive impedance tuning enhance robustness against mass variations, disturbances, and user variability [3,8,9,10,11,12,13,14,15,16,17,18,19,20,21,22].

An LLM primarily extends the high-level layer. Research into planning without task-specific learning has shown that a language model can decompose a high-level command into a sequence of steps, yet a naive plan often fails to align with the set of actions actually available to the robot [24]. SayCan addresses this issue by weighting language-based proposals with the affordance values of learned skills, thereby constraining semantic knowledge by the capabilities of the specific system [23]. PaLM-E incorporates continuous image and state representations into the model, linking language with perception [25]. Other architectures utilize a library of high-level functions and dialogue to generate code [26] or construct policies as programs that invoke explicitly defined control interfaces [27]. The common denominator is grounding the LLM output in a constrained set of executable functions [29,30,31,32,34].

Semantic-level feedback improves planning: Inner Monologue re-triggers reasoning based on success signals, scene descriptions, or human interventions [28]. However, this does not imply that an LLM should close a fast current or thermal control loop. Reviews of foundation models highlight challenges regarding real-time performance, a scarcity of robotics data, uncertainty quantification, and safety guarantees [29,30]. Research on human–robot collaboration demonstrates the benefits of translating diverse utterances into structured task attributes [31] and utilizing function-calling mechanisms [32], while also revealing issues with variable latency and non-determinism. Safety analyses further identify vulnerabilities to ambiguous or adversarial instructions, erroneous grounding, and privacy concerns, as well as the difficulty of online monitoring [24,27,33,35,36,37,38].

For an exoskeleton, the boundary of responsibility should therefore be explicit: the LLM interprets the command and proposes reference parameters, whereas a separate execution layer should enforce the permitted mode, range of motion, speed, acceleration, torque, duration, and temperature limits before passing data to the trajectory generator and actuator-specific controller. The pilot implementation described later uses a narrower prompt-constrained response format and parsing stage; it should not be confused with a formally verified deterministic safety validator.

### 2.6. IT Dimension of the Proposed Architecture

From the perspective of technical informatics, the subject of this work is not only the physical material or actuation mechanism but the computational representation of heterogeneous objects and their integration into a common cyber–physical architecture. The informatics contribution comprises a standardized command interface, modular actuator descriptions, explicit data transformations, a local language-model interface, configurable motion-profile generation, and logging of semantic-layer performance. The architecture is designed so that actuator-specific equations and safety limits can be changed without modifying the user-facing language layer.

Comprehensive comparative validation of such an architecture may include metrics such as RMSE, motion stability, single-iteration time, latency and jitter, memory usage, update frequency, and response to time-budget overruns. For the LLM layer, additional key factors include semantic correctness, schema compliance, and behavior in response to ambiguous commands. However, the scope of this work focuses on architectural coherence and a functional test of the semantic pipeline; thus, the aforementioned metrics serve as criteria for interpreting solutions found in the literature rather than as a set of reported experimental results. The LLM operates outside the real-time loop, while a deterministic validator acts as a safety boundary between interpretation and execution [23,24,25,26,27,28,29,32,34,35,36,37,38]. In this context, SMA and servo drives serve as two demanding design cases for an actuator-independent architecture, rather than objects for a comprehensive quantitative comparison. Their informational value is determined by a shared task representation, modular processing, accurate data logging, and the secure separation of semantics from motion control [3,4,5,6,7,8,9,10,11,12,13,14,15,16,17,18,19,20,21,22].

### 2.7. Research Gap and Positioning of the Study

The existing body of knowledge is extensive but fragmented into loosely connected strands. SMA models describe phase transformation, heat balance, and hysteresis; control research advances compensation, robust methods, and fuzzy/neuro-fuzzy approaches; the exoskeleton literature focuses on interaction, impedance, and actuator selection; meanwhile, work on LLMs primarily addresses planning, manipulation, navigation, and communication. No study within the analyzed corpus was found to integrate these elements into a single architecture or to compare SMA technology against conventional servo-actuation for the same exoskeleton task, shared load, identical trajectory, and uniform set of metrics.

The gap stems not from a lack of individual models, but from a lack of integration and comparable validation. First, publications on SMA and servo-actuation typically employ different systems and operating frequencies, making it unclear whether observed differences arise from the technology, the controller, or the scenario itself. Second, fuzzy and neuro-fuzzy robustness is often evaluated over a limited data range, without simultaneously varying ambient temperature, cooling, resistance, transformation parameters, load, and noise. Third, thermal, energetic, and computational metrics are reported less frequently than tracking error, despite being equally critical for wearable devices. Fourth, LLMs are sometimes presented as general-purpose robot controllers, whereas human-collaborative systems require a formal separation between semantics and execution.

A sound response to this gap is a unified, modular numerical architecture in which the descriptions of the SMA actuator and the servo-actuator remain distinct yet utilize the same task interface. For SMA, this entails explicitly accounting for electro-thermo-mechanical coupling, hysteresis, and temperature limits; for the servo, it involves electrical and mechanical dynamics, current and voltage constraints, friction, and transmission characteristics. Fuzzy and neuro-fuzzy controllers operate within the execution layer, whereas the LLM generates only reference parameters that are passed through a deterministic validator.

This defined scope makes it possible to determine whether systems with radically different dynamics can be encompassed within a single, verifiable task pipeline and where the boundary of the language model’s responsibility should lie. This perspective combines the fidelity of physical representation, software modularity, and the utility of a linguistic interface, without assigning the LLM responsibility for deterministic motion control or predetermining the quantitative superiority of either the SMA or the servo drive.

## 3. Research Methodology and System Implementation

### 3.1. Architecture and Scope

The system was organized as a set of layers with different time scales and responsibilities. The supervisory layer accepts a natural-language command and produces structured target values. The execution layer converts those values into bounded command increments, while the actuator layer represents the technology-specific dynamics. This separation prevents language-model latency and nondeterminism from entering the fast motion loop.

Figure 1 distinguishes the path that was functionally tested on the five-servo hand from the modular comparison layer defined for servo-drive and SMA actuation. At the architecture level, both actuator branches receive the same task reference and should expose comparable outputs. A servo-drive model normally includes electrical and mechanical states, friction, transmission, and saturation. An SMA model additionally requires thermal balance, phase-transformation state, hysteresis, and temperature limits. The common interface therefore standardizes the task description without assuming identical internal physics.

The present experimental implementation covers the LLM-based semantic layer and the two motion-profile generation variants evaluated on the servo-driven hand demonstrator. In parallel, the architecture defines technology-specific locations and interfaces for the numerical dynamic models of the servo drive and SMA actuator, which form the next implementation and validation stage (Figure 1).

The pilot implementation did not use an external position sensor. Consequently, the variable referred to as the current position in the software is the currently commanded pulse width stored by the Servo object, not a measurement of shaft angle. This distinction determines how the fuzzy block should be interpreted in the present study.

### 3.2. Zero-Order Sugeno Motion-Profile Generator

The implemented fuzzy module is a two-input zero-order Sugeno system. Both inputs are expressed in the pulse-width domain. The first input is the difference between the target pulse width and the current commanded pulse width:

*e*(*k*) = *x*_*ref*(*k*) − *x*_*cmd*(*k*) (2)

The second input describes the change in the commanded pulse width between consecutive iterations:

*v*(*k*) = *x*_*cmd*(*k*) − *x*_*cmd*(*k* − 1) (3)

The variable v(k) has the unit µs per iteration and is not an angular velocity. The fuzzy inference stage uses the absolute values |e(k)| and |v(k)|. The sign of e(k) is applied after inference to determine the direction of motion. Five sets were defined for the error (VerySmall, Small, Medium, Large, and VeryLarge) and three sets for the command change (Slow, Medium, and Fast). The error memberships were defined in controller input coordinates as follows: VerySmall, a left-shoulder function through (0,1), (10,1), and (50,0); Small, a triangular function through (20,0), (80,1), and (150,0); Medium, a triangular function through (80,0), (200,1), and (400,0); Large, a triangular function through (250,0), (500,1), and (800,0); and VeryLarge, a right-shoulder function through (600,0) and (1000,1). For the absolute command change, Slow was defined by (0,1), (1,1), and (4,0), Medium by (2,0), (5,1), and (8,0), and Fast by (6,0), (12,1), and (15,1).

Combining the five error sets with the three command-change sets yields 15 rules. For rule i, conjunction is implemented using the minimum operator:

*w*_*i* = *min*(*μ*_*Ai*(|*e*|), *μ*_*Bi*(|*v*|)) (4)

Each consequent z_i is a constant pulse-width increment. The rule base is listed in Table 2.

The crisp output is obtained by weighted averaging:

*s*(*k*) = (*Σ*_*i w*_*i z*_*i*)/(*Σ*_*i w*_*i*) (5)

The value is calculated as a double-precision number, rounded with Math.Round(), and clamped to 1–15 µs per iteration. The upper limit was selected experimentally: larger increments caused blocking or visibly non-smooth servo motion. The lower bound prevents a nonzero error from producing no command change. No adaptive learning was used in the implemented system; it is therefore a fixed-rule zero-order Sugeno generator. A trainable neuro-fuzzy variant remains a direction for future work.

### 3.3. LLM-Based Command Interpretation

The semantic layer was tested on a robotic-hand demonstrator with five servomotors. The user supplied commands such as ‘bend the index finger’ or ‘close hand’. The local LLM translated each command into comma-separated port:angle pairs. The same LLM and prompt were used in both execution variants; only the downstream method used to approach the target pulse width was changed.

The system prompt mapped each finger to a port, limited the requested angle to 0–180°, instructed the model to return only servos that should move, and required the response to contain no explanatory text. Table 3 lists the port mapping. These instructions reduce parser complexity, but they constitute a prompt-based constraint rather than a deterministic safety mechanism.

### 3.4. Software and Hardware Environment

The supervisory application was developed in C# on the .NET platform. System.IO.Ports handled serial communication with the microcontroller, and the OpenAI .NET SDK called a local OpenAI-compatible endpoint provided by LM Studio. The model identifier reported by the environment was google/gemma-4-e4b with Q4_K_M quantization. Inference was performed locally on a Windows 11 Pro computer equipped with an Intel Core Ultra 7 155U processor, 16 GB DDR5 memory, and integrated Intel Graphics using shared system memory (Table 4).

### 3.5. Response Handling and Movement Generation

The application split the LLM response at commas and then separated each item into a port number and an angle. The angle was converted linearly into a pulse width in the 1000–2000 µs range:

*p* = 1000 + *θ*(2000 − 1000)/180 (6)

where θ is the angle in degrees and p is the commanded pulse width in microseconds. Two movement-generation variants were tested. In the first, the target was approached by a constant increment of 2 µs per iteration. In the second, the zero-order Sugeno system selected an increment between 1 and 15 µs per iteration. A 5 ms interval between consecutive command updates was used in both execution variants.

Both execution variants used the same initialization: CurrentPosition = TargetPosition = PreviousPosition = 1500 µs and PreviousVelocity = 0 µs per iteration. They also received the same target pulse-width commands from the semantic layer. In the fixed-step variant, the command changed by 2 µs per iteration. In the Sugeno variant, the CalculateStep(error, velocity) method selected an increment from 1 to 15 µs per iteration, and the sign of the error determined its direction. The resulting commanded value was transmitted in microseconds, while the Arduino Servo library generated the corresponding control pulse subject to the configured limits.

Movement direction and overshoot prevention were implemented independently of the fuzzy inference. For error e(k), the signed increment was limited by the remaining distance to the target, after which the commanded state was clamped to the permissible pulse-width interval:

Δ*x*(*k*) = *clamp*(*sign*(*e*(*k*)) · *s*(*k*), −|*e*(*k*)|, |*e*(*k*)|) (7)

x_cmd(k + 1) = clamp(x_cmd(k) + Δx(k), x_min, x_max)(8)

Thus, sign(e) selects the direction, limiting Δx to |e| prevents overshoot, and the final clamp enforces the configured pulse-width boundaries. Because x_cmd is an internal command state, this logic shapes the command trajectory but does not close a feedback loop around measured shaft position.

### 3.6. Pilot Protocol and Recorded Data

Two movement-generation variants were first checked using the same semantic front-end. In Trial 1, the LLM generated the target servo positions and the application used the fixed 2 µs increment. In Trial 2, the same semantic output was passed to the Sugeno module, which selected the increment from the current error and command-change values. The command “close hand” was used to verify the complete path. The semantic layer was then evaluated using 60 recorded command cases: 27 supported commands for which a target was expected and 33 ambiguous, invalid, unsupported, conflicting, or unrelated commands for which rejection was expected. All cases were executed by the same experimenter using the same prompt, model, and computer configuration. The expected result for each command was defined as either a specific target configuration or rejection.

LM Studio recorded the model response, parsing result, command status, token count, time to first token, generation time, generation speed, and total response time. For each of the 60 cases, semantic correctness was assessed by comparing the parsed target or rejection with the predefined expected result. The physical outcome of supported commands was assessed by direct observation of the actuator. No external position, torque, current, or temperature measurements were recorded. Each command was recorded once in a single configuration; therefore, the reported rates describe this dataset and are not estimates of statistical repeatability.

## 4. Results

### 4.1. Functional Execution of a Natural-Language Command

For the command ‘close hand’, the model returned ‘2:60,3:60,9:60,10:60,11:60’. The response contained all five finger servos and followed the required port:angle schema, allowing the target positions to be parsed without additional natural-language processing.

The target positions were subsequently executed in both movement-generation variants. In the fixed-step variant, each commanded position changed by 2 µs per iteration until the target was reached. In the fuzzy variant, the increment varied between 1 and 15 µs per iteration according to the 15-rule Sugeno system. The available records confirm execution of both data paths but do not provide measured trajectories from which RMSE, settling time, overshoot, or actuator-to-actuator differences could be calculated.

Across 222 servo-level target changes recorded in the available command logs, the adaptive Sugeno profile required 21,703 internal command updates. Reconstructing the same target sequence with the fixed 2 µs increment under matched initial conditions required 38,696 updates. The adaptive profile therefore used 43.9% fewer internal updates. This is an algorithmic measure of convergence of the commanded pulse-width profile; because no external position sensor was used, it does not demonstrate improved physical tracking accuracy, smoothness, settling time, overshoot, or safety.

### 4.2. Evaluation of Supported and Out-of-Scope Commands

The expanded evaluation comprised 60 recorded cases: 27 supported commands and 33 commands that were ambiguous, invalid, unsupported, conflicting, or unrelated to movement. The LLM generated the exact expected target for 25 of the 27 supported commands (92.6%). It correctly rejected 17 of the 33 commands requiring rejection (51.5%). Under a strict command-level criterion, 42 of 60 cases were correct (70.0%). Direct observation confirmed the expected physical outcome in 23 of the 27 supported-command cases (85.2%). Aggregate results are presented in Table 5.

Failures were concentrated in ambiguous or underspecified commands, conflicting targets, and commands that combined valid and invalid elements. Unrelated questions and statements were rejected more consistently, whereas partially valid instructions could lead to execution of only the valid fragment. The 51.5% correct-rejection rate shows that prompt constraints alone are not a deterministic safety mechanism.

### 4.3. Distribution of Local LLM Response Time

Across the 60 recorded test cases, total local response time ranged from 3.73 to 152.88 s. The mean was 25.30 s, the median was 16.71 s, and the 95th percentile was 66.73 s. The distribution is summarized in Table 6.

The broad response-time distribution and its long upper tail are incompatible with direct high-frequency motion control. They are consistent with the intended architectural role of the LLM as an occasional semantic interface whose output is consumed by a separate execution layer operating at a much shorter interval.

## 5. Discussion

The principal result is the verified separation of semantic command interpretation from deterministic movement generation. The same LLM output supplied two execution variants, which demonstrates that the user-facing language interface can remain unchanged while the downstream motion algorithm is replaced. This modularity is the most direct informatics contribution of the study and is relevant to heterogeneous exoskeleton actuation because it prevents actuator-specific variables from propagating into the interaction layer.

The reuse of a single port:angle representation across the two execution variants is consistent with grounding strategies reported in LLM-enabled robotics, where a language model selects among a restricted set of executable functions rather than issuing direct low-level control signals [23,24,25,26,27,28,29,30,31,32,34,35,36,37,38]. The 60-case evaluation extends the evidence beyond a single demonstration. The exact-target rate of 92.6% for supported commands shows that the structured interface can operate reliably within the tested command domain. In contrast, the 51.5% correct-rejection rate and the strict overall result of 70.0% expose a clear weakness for ambiguous, conflicting, and partially invalid inputs. Because each case was recorded once with one prompt, model, and computer configuration, these values characterize the evaluated dataset rather than statistical repeatability. A subsequent benchmark should add repeated paraphrases, controlled prompt and model variations, independent annotation, and a deterministic validation layer.

The implemented Sugeno system should be characterized precisely. Its inputs are derived from commanded pulse-width states and its output selects the next command increment. For the 222 target changes present in the available logs, the adaptive profile required 21,703 internal updates, compared with 38,696 updates for a reconstruction using the fixed 2 µs increment, a reduction of 43.9%. This comparison is quantitative only at the software-command level. Without measured position feedback, the system is not a closed-loop position controller, and the available data cannot establish improved tracking accuracy, smoothness, settling time, overshoot, or safety. Its demonstrated function is adaptive command-profile shaping: large errors can be approached with larger increments, whereas small errors produce smaller increments.

The Sugeno variant also indicates where actuator-independent and actuator-specific processing should be separated. At present, the fuzzy system modifies the increment of a commanded pulse-width trajectory using only command-derived variables. With measured position feedback, the same architectural location could accept tracking error and its derivative; for an SMA actuator, the input vector could additionally include temperature, estimated phase state, resistance, load, or cooling status [142,143,148,150,154]. This extension would not make the controller actuator independent. Rather, it would preserve a common software contract while allowing the rules, state variables, update rate, and safety constraints to remain technology specific. The contribution of the current implementation is therefore the identification and practical test of this interface boundary. Claims regarding smoother motion, improved tracking, or robustness must remain deferred until measured trajectories and repeated disturbance tests are available.

The 51.5% correct-rejection rate confirms that prompt instructions can reduce invalid responses but cannot serve as a deterministic validator. The model sometimes accepted ambiguous requests or executed the valid portion of a command that also contained an invalid port, angle, or conflicting target. Before use in a human-interacting device, the parser should atomically reject any command that violates the allowed-port list, numeric ranges, command cardinality, consistency rules, communication timeouts, or actuator-specific safety limits. For an SMA branch, these checks must also include current, temperature, stress, duty cycle, and cooling-state constraints.

The mean response time of 25.30 s, the 95th percentile of 66.73 s, and the maximum of 152.88 s further support keeping the LLM outside the real-time loop. The architecture does not require the semantic layer to run at the servo update rate; it only requires a stable structured interface between infrequent user commands and the execution process. Faster or smaller models may reduce this latency, but the division of responsibilities should be preserved even if inference becomes substantially faster.

For SMA actuation, the literature consistently identifies coupled thermal dynamics, hysteresis, limited cooling rate, and parameter variability as central modelling and control challenges [165,166]. Reviews and exoskeleton-oriented studies also show that low mass, silent operation, mechanical compliance, and compact integration may justify the technology in selected wearable applications, provided that bandwidth and thermal safety are treated explicitly [167,168]. These findings support the architecture-level inclusion of an SMA branch but do not substitute for actuator-specific equations, calibrated parameters, or experimental trajectories.

The present validation covers the implemented semantic and servo motion-profile path. The servo-drive and SMA dynamic-model modules remain at the architectural specification stage; consequently, the comparison branch defines a roadmap for subsequent implementation rather than an experimentally validated actuator comparison.

The current evidence therefore does not demonstrate that SMA actuators outperform servo drives, nor that the fuzzy algorithm compensates SMA hysteresis. It establishes a common task pipeline and identifies the information that an actuator-specific implementation must expose. A complete comparative study would require identical reference profiles and loads, measured position and thermal states, repeated trials, and metrics such as RMSE or MAE, phase lag, settling time, energy, peak power, saturation duration, and computation time.

The neuro-fuzzy concept should likewise be treated as future work until a training set, architecture, adaptation rule, and independent validation protocol are available. Its potential value lies in adapting membership functions or rule consequents to changing actuator conditions; no result in the present dataset supports a claim of superiority over the implemented fixed-rule Sugeno system.

## 6. Conclusions

A layered architecture was developed to separate natural-language interpretation, structured target generation, motion-profile generation, and actuator-specific execution. This organization permits the semantic interface to remain independent of whether the task is ultimately executed by a conventional servo drive or an SMA-based module.

The implemented pilot verified the complete path from an English command to five servo target positions. The same LLM front-end was used with a constant 2 µs per-iteration increment and with a 15-rule zero-order Sugeno system selecting increments from 1 to 15 µs per iteration. For 222 recorded target changes, the adaptive profile required 21,703 internal command updates, compared with 38,696 updates in the fixed-step reconstruction, a reduction of 43.9%. In the absence of an external position sensor, this result describes only the software command profile, and the Sugeno variant is appropriately characterized as a fuzzy motion-profile generator rather than a closed-loop position controller.

In the 60-command evaluation, the exact expected target was generated for 25 of 27 supported commands (92.6%), while 17 of 33 invalid or unsupported commands were correctly rejected (51.5%). The strict overall result was 42 of 60 cases (70.0%), and direct observation confirmed the expected physical outcome in 23 of 27 supported cases (85.2%). The lower rejection performance prevents treating the LLM as a safety mechanism. Independent range, schema, consistency, timeout, and actuator-safety checks remain necessary before deployment in an exoskeleton.

The mean local response time of 25.30 s and the 95th percentile of 66.73 s confirm that the LLM should remain in the supervisory layer rather than the real-time motion loop. The available results confirm the feasibility of the modular data path. Quantitative claims about trajectory-tracking improvement or the relative performance of SMA and servo actuation require the next validation stage, in which the defined technology-specific modules will be populated with calibrated dynamic models and tested using common reference tasks, measured states, and consistent performance metrics. The framework therefore provides a defensible and experimentally informed basis for that comparison.

## Figures and Tables

**Figure 1:**
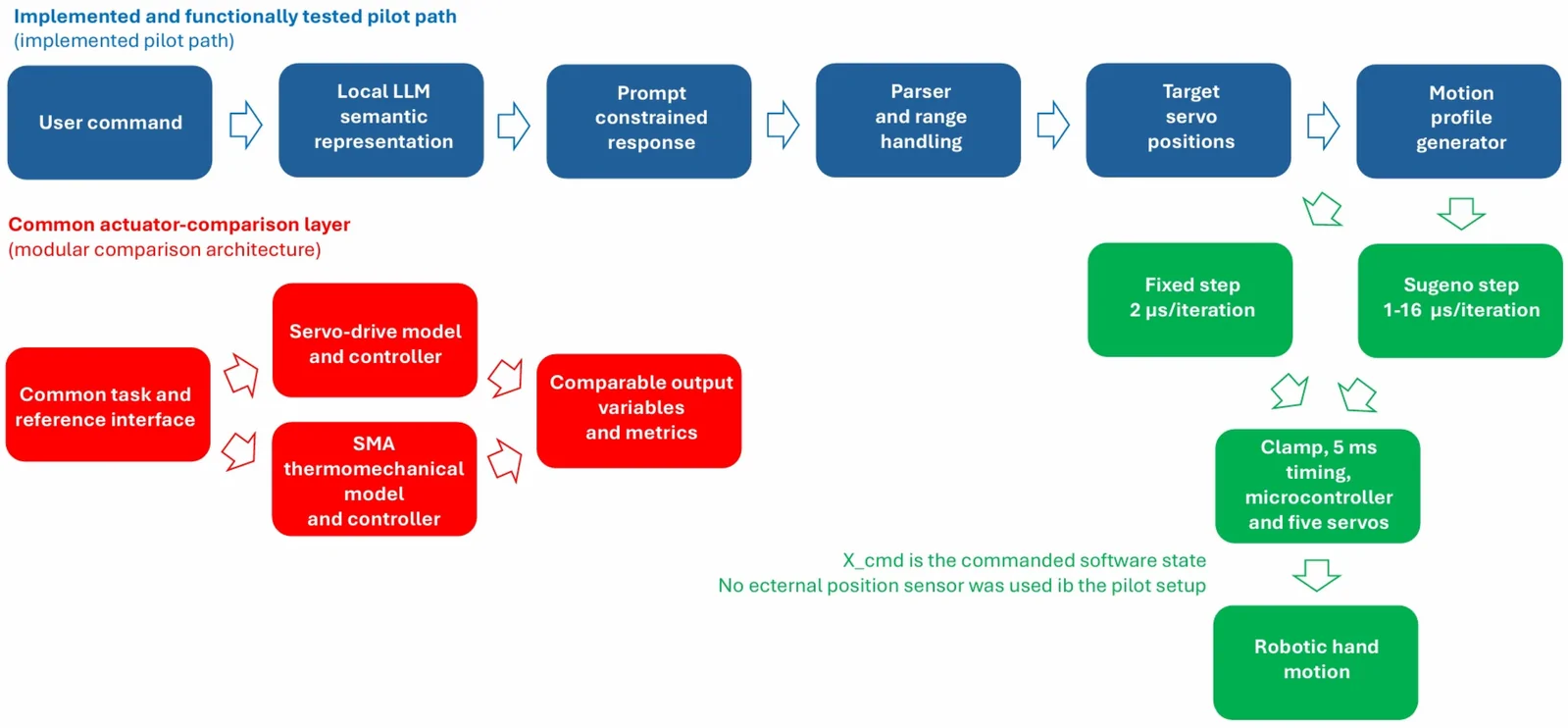
Layered architecture. Solid connections denote the implemented pilot path; dashed connections denote the proposed comparison layer for future actuator-specific numerical models and controllers.

**Table 1:** Literature-derived variables and constraints required for a future comparison of SMA actuators and conventional servo drives.

Comparison Dimension	SMA Actuator: Variable or Constraint	Conventional Servo Drive: Variable or Constraint
Actuation path	Electrical input, Joule heating, phase transformation, strain or force, and transmission	Electrical input, electromagnetic torque, shaft motion, and gearbox or transmission
Dynamic states to report	Temperature, phase fraction, stress or strain, electrical resistance, and cooling state	Current, speed, position, torque, and mechanical load
Technology-specific uncertainties	Hysteresis, convection, ambient temperature, load dependence, and fatigue	Friction, backlash, electrical parameters, inertia, and transmission compliance
Potential wearable benefits to verify	Low active-element mass and volume, quiet operation, and flexible or textile integration	High bandwidth and repeatability, with mature sensing and drive electronics
Primary constraints	Cooling rate, operating temperature, limited wire stroke, and cycle life	Gearbox mass, reflected impedance, acoustic noise, and mechanical complexity
Applicable control structure	Hysteresis compensation with feedback; adaptive, robust, fuzzy, or neuro-fuzzy control	Cascaded current-speed-position loops; PI/PID, sliding-mode, fuzzy, or model predictive control
Safety variables	Temperature, current, stress, transformation range, and duty cycle	Torque, current, speed, position, and interaction energy

**Table 2:** Rule base of the zero-order Sugeno system; entries are pulse-width increments in µs per iteration.

Error Set	Slow	Medium	Fast
VerySmall	1	1	1
Small	3	2	1
Medium	7	6	4
Large	11	9	7
VeryLarge	15	13	10

**Table 3:** Port mapping used in the robotic-hand pilot setup.

Manipulator Component	Port
Index finger	2
Middle finger	3
Thumb	9
Ring finger	10
Little finger	11

**Table 4:** Software and local-inference configuration used in the pilot study.

Item	Configuration
Host application	C#/.NET
Serial interface	System.IO.Ports
LLM client	OpenAI .NET SDK
Local inference server	LM Studio 0.4.24; OpenAI-compatible endpoint at http://localhost:1234/v1 (accessed on 30 September 2026)
Model	google/gemma-4-e4b; Q4_K_M
Context length	8192 tokens
GPU offload	29 layers
Flash Attention	Enabled
Sampling	Temperature 1.0; Top P 0.95
Computer	Intel Core Ultra 7 155U; 16 GB DDR5; integrated Intel Graphics; Windows 11 Pro
Controller board	DFRobot Romeo V2; ATmega32U4, 16 MHz; 5 V logic
Serial configuration	9600 baud; 8 data bits; no parity; 1 stop bit (8N1)

**Table 5:** Aggregate results of the 60-command evaluation.

Evaluation Subset	Cases (n)	Correct Outcomes (n)	Rate
Supported commands: exact target generation	27	25	92.6%
Invalid or unsupported commands: correct rejection	33	17	51.5%
All commands: strict semantic outcome	60	42	70.0%
Supported commands: observed physical outcome	27	23	85.2%

**Table 6:** Distribution of total local LLM response time across 60 recorded test cases.

Metric	Value
Recorded test cases	60
Mean total response time	25.30 s
Median total response time	16.71 s
95th percentile	66.73 s
Minimum	3.73 s
Maximum	152.88 s

## Data Availability

The original contributions presented in this study are included in the article. Further inquiries can be directed to the corresponding author.

## Abbreviations

The following abbreviations are used in this manuscript:

1D-CNN	One-dimensional convolutional neural network
AI	Artificial intelligence
ANFIS	Adaptive Neuro-Fuzzy Inference System
BLDC	Brushless direct-current motor
FEM	Finite element model
LLM	Large language model
LSTM	Long Short-Term Memory
ML	Machine learning
PI	Proportional–integral
PID	Proportional–integral–derivative
PMSM	Permanent magnet synchronous motor
PWM	Pulse-width modulation
SMA	Shape-memory alloy
